# Use of dual antiretroviral therapy in individuals with different serological patterns for hepatitis B: What are the risks? What are the clinical implications?

**DOI:** 10.1111/hiv.70063

**Published:** 2025-06-16

**Authors:** Arda Kaya, Dominik Benke, Tracy Swan, Sven Breitschwerdt, Jan‐Christian Wasmuth, Christoph Boesecke, Jürgen Kurt Rockstroh

**Affiliations:** ^1^ Department of Infectious Diseases and Clinical Microbiology Ege University Faculty of Medicine İzmir Turkey; ^2^ Department of Medicine I University Hospital Bonn Bonn Germany; ^3^ GlobalHealth Consultant Barcelona Spain

**Keywords:** dual ART, hepatitis B, HIV

## INTRODUCTION

With the introduction of the first registered long‐acting two‐drug antiretroviral therapy cabotegravir/rilpivirine (CAB/RPV‐LA), a growing number of people living with HIV have been switched over to this new treatment option, often upon patient request, to help overcome HIV stigma and pill fatigue. Transitioning off a mostly tenofovir‐based treatment, however, comes at the risk of hepatitis B virus (HBV) infection or reactivation. Besides CAB/RPV‐LA, there has been increasing interest in two‐drug regimens (2DR) without anti‐HBV activity, including daily, oral weekly, or long‐acting injectable therapies. In view of these trends, we discuss potential risks and clinical implications here (key messages summarized in Table 1).

**TABLE 1:** Key messages.

Primary HBV infections in people living with HIV switching over from tenofovir‐containing regimens to 2DR without HBV activity are preventable. Vaccinate before switching over, and ensure adequate vaccine response according to the respective guidelines (e.g. [1, 2]).
Antiretroviral treatment trials are increasingly evaluating tenofovir‐free regimens and will continue to report cases of primary HBV infections, unless vaccination of susceptible participants is enforced by protocol.
People living with HIV with isolated anti‐HBc are at risk of HBV reactivation after switching away from tenofovir‐containing ART and will therefore require close monitoring. Individual risk factors for HBV reactivation in this context need to be further evaluated, as well as the clinical relevance of low‐level HBV viraemia without elevated transaminases or detectable HBs antigen.
A documented “serological cure” (anti‐HBc+, anti‐HBs+) does not safely rule out HBV reactivation in people living with HIV. A rise in transaminases after switching over to 2DR without HBV activity should prompt HBV diagnostics.

Abbreviations: 2DR, dual drug regimen; ART, antiretroviral therapy; HBc, hepatitis B core antigen; HBs, hepatitis B surface antigen; HBV, hepatitis B virus; PLWH, people living with HIV.

## ROLE OF TENOFOVIR

Due to shared transmission routes, HBV/HIV coinfection is common. The European AIDS Clinical Society (EACS) guidelines recommend tenofovir‐containing antiretroviral therapy (ART) for people with HBV/HIV coinfection. Tenofovir (given as one of the available prodrugs TDF or TAF) not only treats existing hepatitis B, but also protects against acquiring HBV infection [3, 4]. The protective effect has also been shown for men who have sex with men under daily HIV pre‐exposure prophylaxis [5]. Lamivudine has also been shown to offer some protection against incident HBV infection, albeit to a lesser extent [3, 4]. Ongoing risk behaviour, lack of implementation of EACS vaccination recommendations [6] and insufficient response to HBV vaccines due to HIV‐associated immunodeficiency [7] put people living with HIV at an increased risk of acquiring HBV infection unless they are on a tenofovir‐based regimen. Conversely, tenofovir‐based ART should not lead clinicians to neglect HBV vaccination, which remains the first choice in preventing hepatitis B.

## SWITCHING TO 2DR IN PEOPLE LIVING WITH HIV WITHOUT PRIOR HBV INFECTION

There are various reasons why people living with HIV or their physicians might want to change to dual therapy without HBV activity. Apart from relatively rare cases when certain ARTs are necessary for salvage therapy, there is usually no great urgency to switch over to a regimen that is not active against HBV, allowing time to perform HBV serological tests and, if needed, vaccination. Response to the vaccine should be assessed to ensure that protective anti‐HBs levels are present. Development of a novel HBV vaccine using the Toll‐like receptor agonist CpG instead of classic alum‐based adjuvants has significantly improved vaccine responses in people living with HIV, thus providing robust HBV prevention without tenofovir‐based ART in countries where this vaccine is available [8, 9]. Repeat vaccinations or higher dosages for yeast‐based vaccines represent a well‐established alternative in all settings [1]. Nevertheless, recently published data from trials evaluating 2DR still show cases of HBV infection in the 2DR study arms. For instance, a trial on switching over to doravirine/islatravir (DOR/ISL) from bictegravir/emtricitabine/tenofovir reported two cases of acute HBV infection through Week 48 in the DOR/ISL group; data on vaccination history were not provided, but baseline serology did not show anti‐HBs or anti‐HBc antibodies [10]. In a phase II study on weekly oral ISL plus lenacapavir (LEN), one of the 52 patients in the ISL/LEN arm discontinued the study due to acute hepatitis B [11]. This person also had a negative serology for anti‐HBc and anti‐HBs at baseline, with no recorded history of non‐response to HBV vaccination (J. Hindman, personal communication). Although study protocols on new 2DR often advise recommending HBV vaccination for study participants who were found susceptible to HBV infection at screening (anti‐HBs negative), neither vaccination nor control of anti‐HBs titres after vaccination are commonly considered mandatory for enrolment.

## SWITCHING OVER TO 2DR IN PEOPLE LIVING WITH HIV WITH PRIOR HBV INFECTION

People living with HIV with active or occult HBV infection should remain on ART that includes tenofovir. If HBs antigen or HBV‐DNA are positive, 2DR without HBV activity should not be used. Less obvious are cases with isolated anti‐HBc antibodies. Does the low risk of HBV reactivation in these patients justify depriving them of the potential benefits of 2DR? If HBV‐DNA is negative, occult hepatitis B is less likely, but cannot be ruled out with certainty, especially if the result was obtained under tenofovir‐containing ART and not before ART initiation. Reported frequencies of HBV reactivation after switching to 2DR without HBV activity range from 0% in a Spanish cohort [12], 1.6% in a US cohort [13], to 10% in a randomized trial in Cameroon [14]. More recently, the above‐mentioned study on DOR/ISL also reported two cases where a low level of HBV‐DNA became detectable after switching over (one being anti‐HBc+/anti‐HBs+ at baseline, the other anti‐HBc+/anti‐HBs−) [10]. Of note, a history of anti‐HBs antibodies in people living with HIV with HBV infection under tenofovir‐containing ART does not guarantee immunity to HBV, as recently shown in a case report: The patient's anti‐HBs titres had declined and become undetectable under a tenofovir‐containing ART before being switched over to a 2DR. After the switchover, HBs antigen and HBV‐DNA became detectable again [15]. Generally, the risk of reactivation is increased in the context of immunosuppression, with chemotherapy or organ transplantation being known risk factors [16]. We therefore believe that patient education on the risk of HBV reactivation should be part of the shared decision‐making process and that individual as well as regional risk factors should be taken into account before switching over to ART. After switching over, new risk factors, such as the initiation of immunosuppressive therapy, should be regularly reassessed to determine whether a return to tenofovir‐containing ART is required. If possible, HBV parameters should be closely monitored after switching over, and any rise in transaminase levels should prompt further testing to rule out HBV reactivation. In settings where HBV‐DNA testing is not available, the decision on reintroducing tenofovir can be based on testing of HBs antigen alone, which is cheap and widely available.

## CONCLUSION

The risk of HBV infections in people living with HIV has long been known, and will gain importance with the rise of new HIV treatment regimens without HBV activity. Highly effective vaccines can be used to help prevent primary HBV infection and should be administered *before* transitioning off an HBV‐active ART. Open questions remain with regard to the risk of (and risk factors for) reactivation in the context of switching from tenofovir‐containing regimens, and the clinical significance of low‐level HBV viraemia without elevated transaminase levels or detectable HBs antigen. While guidelines mention HBV reactivation screening following ART modification [2, 17], a detailed monitoring schedule defining parameters and timeline remains undeveloped and is beyond the scope of this viewpoint. For now, an increased vigilance after switching over is certainly advisable.

## AUTHOR CONTRIBUTIONS

Arda Kaya, Dominik Benke, and Jürgen Kurt Rockstroh drafted the manuscript, which was actively reviewed and commented on by Tracy Swan, Sven Breitschwerdt, Jan‐Christian Wasmuth, and Christoph Boesecke.

## CONFLICT OF INTEREST STATEMENT

JKR has received honoraria from Abbvie, Gilead, Janssen, MSD, and ViiV, for consulting or speaking at educational events. CB has received honoraria from Abbvie, Bavarian Nordic, Gilead, Janssen, MSD, Pfizer, and ViiV, for consulting or speaking at educational events. AK, DB, TS, SB and JCW have no conflicts to report.
